# Micromechanical behavior of the apple fruit cuticle investigated by Brillouin light scattering microscopy

**DOI:** 10.1038/s42003-025-07555-5

**Published:** 2025-02-04

**Authors:** Timm Landes, Bishnu P. Khanal, Hans Lukas Bethge, Tina Lehrich, Maximilian Seydi Kilic, Franz Renz, Miroslav Zabic, Moritz Knoche, Dag Heinemann

**Affiliations:** 1 Hannover Centre for Optical Technologies, Leibniz University Hannover, Nienburger Straße 17, 30167 Hannover, Germany; 2 Cluster of Excellence PhoenixD, Leibniz University Hannover, Welfengarten 1a, 30167 Hannover, Germany; 3 Institute of Horticultural Production Systems, Leibniz University Hannover, Herrenhäuser Straße 2, 30419 Hannover, Germany; 4 Institute of Cell Biology and Biophysics, Leibniz University Hannover, Herrenhäuser Straße 2, 30419 Hannover, Germany; 5 Institute of Inorganic Chemistry, Leibniz University Hannover, Callinstraße 3-9, 30167 Hannover, Germany

**Keywords:** Nanoscale biophysics, Biophysical methods, Plant physiology, Optical spectroscopy

## Abstract

The cuticle is a polymeric membrane covering all plant aerial organs of primary origin. It regulates water loss and defends against environmental stressors and pathogens. Despite its significance, understanding of the micro-mechanical properties of the cuticle (cuticular membrane; CM) remains limited. In this study, non-invasive Brillouin light scattering (BLS) spectroscopy was applied to probe the micro-mechanics of native CM, dewaxed CM (DCM), and isolated cutin matrix (CU) of mature apple fruit. The BLS signal arises from the photon interaction with thermally induced pressure waves and allows for imaging with mechanical contrast. The derived loss tangent showed significant differences with wax extraction from the CM and further with carbohydrate extraction from the DCM, consistent with tensile test results. Spatial heterogeneity between anticlinal and periclinal regions was observed by BLS microscopy of CM and DCM, but not in CU. The key conclusions are: (1) BLS is sensitive to micro-mechanical variations, particularly the strain-stiffening effect of the cutin framework, offering insights into the CM’s micro-mechanical behavior and underlying chemical structures; (2) CM and DCM exhibit spatial micro-mechanical heterogeneity between periclinal and anticlinal regions.

## Abstract (web summary)

A Brillouin spectroscopy study of the apple fruit cuticle indicates spatial micro-mechanical inhomogeneities exist between the periclinal and anticlinal regions.

## Introduction

The cuticle (cuticular membrane; CM; Supplementary Fig. 1) is a non-living polymeric membrane that covers the primary surfaces of aerial organs of all terrestrial plants, including fruit^1^. It consists of three main components: cutin, wax, and polysaccharides^1,2^. The CM serves several important protective functions^2,3^, which include the barrier function against excessive water transport^4–6^, pathogen infestation, insect and other physical damages^7–9^, and shielding from harmful ultraviolet radiation^1^.

During fruit growth and development, the cuticle is subjected to mechanical stress from the expanding fruit surface^10^. The fruit needs to preserve the cuticle’s integrity and compensate for its expansion throughout fruit development^11–13^, as any damage to the cuticle can result in impaired barrier function^14,15^. To prevent failure, apple fruits synthesize and deposit additional cutin and wax. The newly synthesized cutin is deposited on the inner surface of the existing cuticle^11,13,16^, while wax fills the intermolecular spaces of the cutin-carbohydrate network^17^. This interaction of the components leads to a plastic fixation of the elastic deformation of the cuticle^12,13,18,19^. In other words, the elastic strain is fixated by the filling effect of the wax^18,20^. Extraction of wax from the CM or removal of new cutin layers from the inner side of the CM results in the release of deformation and the stiffness of the apple fruit CM decreases^13,18^. The carbohydrates present in the cuticle are part of the outer epidermal cell walls that penetrate the cutin network^21^. They also play an important role in cuticle biomechanics^2,22–25^. The macro-mechanical properties of the cuticle have been investigated quite significantly utilizing uniaxial tensile test^17,18,22,24–27^ and dynamic mechanical analysis^23^, micro-mechanical investigations only have been recently reported^28^.

The authors used atomic force microscopy (AFM) to map the Young’s modulus across the surface of isolated tomato skin samples and on cross-sectional histological cuts. However, to accomplish this, the wax present in the cuticle had to be removed. The use of contact techniques in a waxy environment can lead to contamination of the tip and the optical path or heat generation in the sample. Although some of the difficulties can be overcome using coated AFM tips and careful parameter selection^29^, the methods remain constrained to the assessment of the prepared, dewaxed sample surface. In addition, the extraction of the mechanical parameters is based on the calculation of a mechanical model.

In the last decade, Brillouin light scattering (BLS) microscopy has been described as a novel, high-resolution, non-contact, marker-free tool to shed new light on plant mechanobiology^30–37^. In BLS spectroscopy, incident photons scatter on thermally induced density fluctuations within the sample. Since the density fluctuations travel at the speed of sound, a tiny frequency shift ( < 1 cm^-1^) is induced to the scattered photon, which is proportional to the acoustical speed. From this shift and the corresponding linewidth information regarding the longitudinal modulus (i.e. sample compressibility) can be derived. This, however, requires knowledge about the refractive index of the sample and the density. In addition, the loss tangent, a measure for attenuation of mechanical waves, can be derived. This is independent on refractive index and density of the sample^35,38^.

As BLS spectroscopy relies only on optical phenomena, it can be coupled to a confocal microscope, allowing microscopical three-dimensional imaging with mechanical contrast. Doing so, the spatial resolution of roughly one micrometer can be achieved (*λ*_0_ = 532 nm; NA = 0.4) over a wide field of view, while the smallest acoustical wavelength detectable is approximately 200 nm (${\Lambda} ={\lambda }_{0}/2n$; *n* ≈ 1.5)^38^.

In this study, we employed BLS spectroscopy to examine the micro-mechanical properties of the apple cuticle, which has high wax content^26^. We compared the results from BLS spectroscopy to those obtained by conventional macro-mechanical testing procedures^24^. Using this approach, we further investigated micro-mechanical inhomogeneities of the CM and the impact of its chemical constituents thereon, by removing the waxes (dewaxed CM, DCM) and treating the DCM with hydrochloric acid, releasing the carbohydrates fraction from the cutin network (CU). We hypothesize that the presence of waxes and the carbohydrates fraction significantly influence the micro-mechanical properties of the apple cuticle reflected by BLS measurements, and that their impact shows spatial variation across the cuticle.

## Results

### Brillouin light scattering spectroscopy shows mechanical changes upon wax and carbohydrate removal

**Fig. 1:**
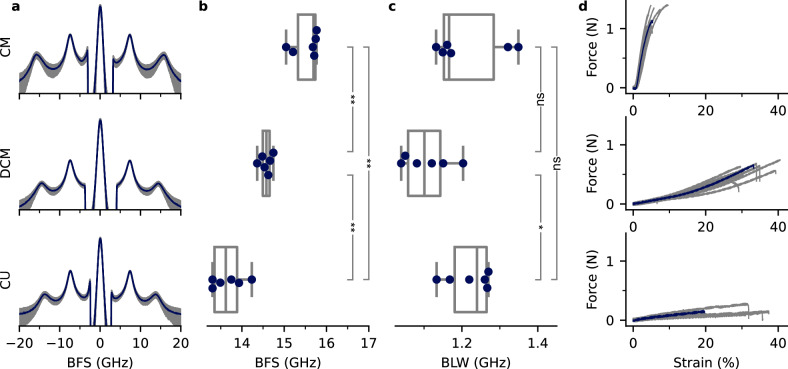
Mechanical characterization of the cuticular membrane. **a** Averaged Brillouin spectra and standard deviation of the cuticular membrane (CM), dewaxed CM (DCM), and the acid resistant fraction of DCM (*cutin matrix*, CU) showed the water peak at around 7.5 GHz and the sample peak at around 15 GHz. Signal intensity was plotted logarithmically. Box plots of averaged Brillouin frequency shift (BFS; **b**) and Brillouin line width (BLW; **c**) of the extracted spectra peak of CM, DCM, and CU. Each sample point is the average over a 2D scan with 900 data points. **d** Force-strain diagrams of appropriately treated samples obtained from uniaxial standard tensile tests. One representative curve is shown in blue. * and ** denote *p* < 0.05 and *p* < 0.01 using the Mann-Whitney-U test, ns indicates non-significant differences.

BLS data was acquired for CM, DCM, and CU. The representative Brillouin spectra are shown in Fig. 1a. Beside the Rayleigh scattered light, two distinct peaks are visible. The peak at 7.5 GHz is related to the deionized water^39^, while the peak between 12 to 16 GHz is attributed to the hydrated cuticle samples. The extracted sample BFS and BLW were then averaged for each sample (900 sampling points). The corresponding averaged BFS and BLW for the samples are shown in Fig. 1b, c. Upon wax removal, the BFS decreased significantly (*p* < *0.01*, Mann-Whitney-U). The carbohydrate (CHO) extraction (mainly polysaccharides from the outer epidermal cell walls) led to a further reduction in BFS, significantly lower than the DCM.

The BLW showed a decrease *(p* = *0.06)* upon wax extraction and increased significantly after the CHO extraction *(p* < *0.05)*. There was no significant difference between the BLW in CM and CU.

The averaged loss tangent for CM was determined to ${\delta }_{{\mbox{CM}}}=0.982\pm 0.104$ and for DCM ${\delta }_{{\mbox{DCM}}}=0.955\pm 0.088$. This reduction was significant under Mann-Whitney-U test (*p* < 0.0001). The loss tangent for CU increased to ${\delta }_{{\mbox{CU}}}=1.122\pm 0.162$, significantly higher then CM and DCM (both *p* < 0.0001, Mann-Whitney-U test).

Uniaxial force-strain diagrams were recorded for CM, DCM, and CU ($N=12$ each) to compare with the BLS data. Figure 1d shows representative graphs with varying force-strain behavior. The maximum slope of the curve decreased after wax extraction of the CM and was further reduced after CHO extraction of the DCM. There was a marked decrease in averaged stiffness $S$ (determined as the slope of the curve in the elastic region) and tensile strength ${F}_{\max }$ on wax extraction of the CM and a further decrease after CHO extraction of DCM. Conversely, the averaged maximum measured strain ${\varepsilon }_{\max }$ increased sharply on wax extraction and decreased slightly on CHO extraction (Table 1).

**Table 1:** Comparison of micro- and macro-mechanical measured variables

Sample type	BLS parameters		Macro-mechanical properties		
	BFS (GHz)	BLW (GHz)	*S* (N)	*F*_max _(N)	*ε*_max_ (%)
**CM**	$15.52\,\pm \,0.29$	$1.21\,\pm \,0.09$	$37.74\,\pm \,3.61$^a^	$1.17\,\pm \,0.16$^a^	$4\,\pm \,1$^a^
**DCM**	$14.57\,\pm \,0.13$	$1.11\,\pm \,0.06$	$2.30\,\pm \,0.43$^b^	$0.59\,\pm \,0.10$^b^	$28\,\pm \,4$^b^
**CU**	$13.67\,\pm \,0.34$	$1.22\,\pm \,0.05$	$0.26\,\pm \,0.19$^c^	$0.15\,\pm \,0.05$^c^	$23\,\pm \,10$^c^

Brillouin light scattering (BLS) parameters and macro-mechanical properties (mean ± SD) of ‘Idared’ apple fruit cuticular membrane (CM), dewaxed CM (DCM) and the acid resistant fraction of DCM (cutin matrix, CU) (*N* = 12 uniaxial tensile test and *N* = 6 BLS). Groups with different letters in the macro-mechanical properties denote *p* < 0.01 (Mann-Whitney-U test).

### Wax crystallinity affects Brillouin light scattering parameters of the apple cutin polymer

**Fig. 2:**
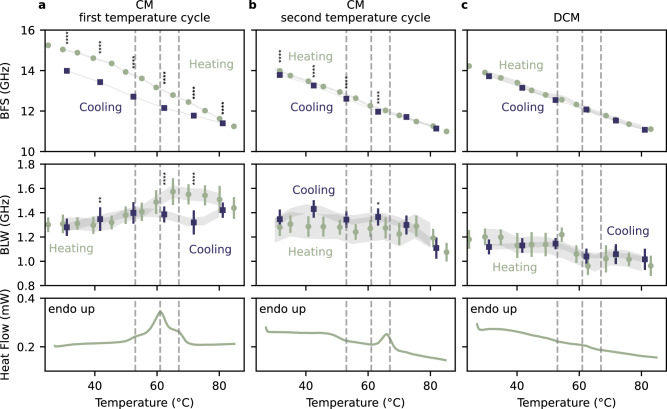
Thermal relaxation processes of the cuticular membrane. Effect of heating and subsequent cooling of isolated and hydrated cuticular membrane (CM; **a, b**) and dewaxed CM (DCM; **c**) of ‘Idared’ apple on Brillouin frequency shift (BFS, average & standard deviation), Brillouin line width (BLW, average & standard deviation), and heat flow (differential scanning calorimetry; DSC) after **a** CM first temperature cycle and, **b** second temperature cycle. Additionally, the effect was investigated in DCM (**c**) after first temperature cycle. DSC thermographs show the melting temperature of the embedded wax. The distinct peaks in heat flow of CM wax (53* ± *1 °C, 61 ± 1 °C, and 67 ± 1 °C) are marked with the dotted lines. *, **, ***, **** denote *p* < 0.05, *p* < 0.01,* p *< 0.001, and *p* < 0.001 using mixed linear-effect model and post-hoc Tukey analysis.

CM and DCM were heated and cooled subsequently. The temperature dependence of the BFS and BLW are shown in Fig. 2. The BFS of CM exhibited a consistent monotonic decrease with increasing temperature up to 80 °C. The decrease rate increased at approximately 49 °C (Supplementary Fig. 2a) Afterwards, the samples were gradually cooled down to room temperature. During cooling, the BFS showed a corresponding monotonic increase. The BFS during the cooling phase was significantly lower than in the heating phase (Fig. 2a). The maximum hysteresis was measured to be 1.2 GHz. In contrast to heating, the BFS increase rate increased at approximately 64 °C during cooling (Supplementary Fig. 2a).

The BLW of the CM revealed no significant change up to approximately 50 °C. However, as temperature further increased, the BLW increased, reaching a maximum at approximately 62 °C. This coincides with the major peaks (53 ± 1 °C, 61 ± 1 °C, and 67 ± 1 °C) in the DSC thermographs (Fig. 2a). The peaks of the thermograph indicate the melting of the wax^18^. Following wax melting, the BLW decreased again with increasing temperature up to 80 °C. During the cooling phase, the BLW decreased as the temperature decreased until it reached 53 °C, showing a significant different trend. After that, it increased until it returned to its initial value at room temperature (Fig. 2a).

A second temperature cycle of the previously heated sample did show a reduced but still significant hysteresis in the BFS (Supplementary Fig. 2b) and a similar monotonic decrease with increasing temperature to the first heating phase. The BLW remained constant until 70 °C (Fig. 2b). At higher temperatures the BLW was significantly reduced. During cooling, the BLW was significantly higher from 70 °C to 40 °C. The DSC thermograph had only a single peak at approximately 62 °C.

The DCM showed a similar behavior in BFS to the CM after its second heating cycle. No significant hysteresis was found in DCM between heating and cooling phase (Fig. 2c). The BLW was slightly reduced compared to the second heating cycle of the CM and showed no significant difference between the heating and cooling phases. A decrease was observed at around 60 °C. Correspondingly, the DSC thermograph showed a weak peak at the same temperature.

### Brillouin microscopy maps display spatial heterogeneities of the apple cutin polymer matrix along its surface

**Fig. 3:**
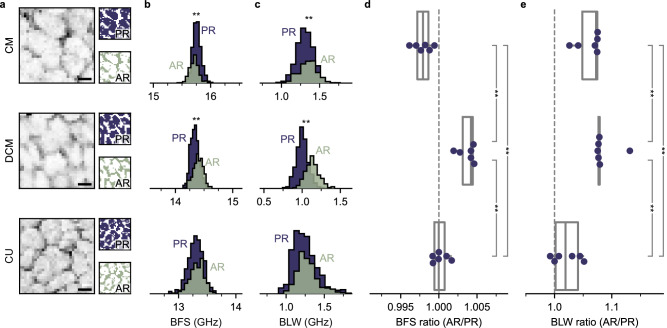
Spatial heterogeneities within the cuticular membrane visible with Brillouin light scattering. **a** Brillouin intensity maps and the calculated masks for periclinal regions (PR) and anticlinal regions (AR) of the cuticular membrane (CM), dewaxed CM (DCM), and the acid-resistant fraction of DCM (cutin matrix, CU). Scale bars denote 20 µm. Representative normalized Brillouin frequency shift (BFS) **b** and Brillouin line width (BFS) c for AR and PR. Diagrams in **b** and **c** are scaled identically for CM, DCM, and CU, but have a different offset. Calculated ratio of averaged BFS **d** and BLW **e** for AR and PR. * and ** denotes *p *< 0.05 and *p* < 0.01 using the Mann-Whitney-U test.

The Brillouin surface maps revealed the structural imprints of the epidermal cells with strong BLS intensity in the anticlinal regions and weak BFS signal intensity in the periclinal regions (Fig. 3a and Supplementary Fig. 3). To further analyze the heterogeneities, a local adaptive threshold method was developed and applied to the BLS intensity maps (Fig. 3a and Supplementary Fig. 4-6). The resulting masks for anticlinal and periclinal regions were then applied to the BFS and BLW scans (respectively shown in Fig. 3b and c).

For CM, the anticlinal regions showed a significantly smaller BFS compared to the periclinal regions (Fig. 3b). The BLW for the anticlinal regions was higher compared to the periclinal regions (Fig. 3c). In DCM, both the BFS and the BLW of the anticlinal regions were higher than that of the periclinal regions. CU, the BFS and the BLW were similar for periclinal and anticlinal regions.

The ratios between anticlinal regions and periclinal regions were calculated for the BFS and the BLW (Fig. 3d and e). The BFS ratio of the CM was found to be significantly lower compared to the DCM and CU. Heating of the CM did not affect the ratio (Supplementary Fig. 7). Further, the BFS ratio of DCM was significantly higher than the BFS ratio of the CU (Fig. 3d). The BLW ratio behaved similarly (Fig. 3e). The BLW ratio of CM and DCM were similar but significantly higher than CU (*p* = *0.005*).

The loss tangents of the PR and AR for CM, DCM, and CU were calculated (Table 2). Differences between AR and PR were significant for all CM and DCM (*p *< 0.0001) and only for 3 of 6 CU (*p* < 0.05). The differences between CM, DCM and CU were all significant.

**Table 2:** Comparison of the loss tangent between anti- and periclinal regions

Sample type	Loss tangent	
	AR	PR
CM	$1.025\pm 0.105$^a^	$0.962\pm 0.098$^b^
DCM	$1.004\pm 0.084$^c^	$0.928\pm 0.081$^d^
CU	$1.131\pm 0.123$^e^	$1.104\pm 0.137$^e^

Loss tangent for anticlinal (AR) and periclinal regions (PR) (mean ± SD, *N* = 6) of ‘Idared’ apple fruit cuticular membrane (CM), dewaxed CM (DCM) and the acid-resistant fraction of DCM (cutin matrix, CU). Groups with different letters in the macro-mechanical properties denote *p* < 0.0001 (Mann-Whitney-U test).

### Assessment of the refractive index distribution in apple cuticular membranes

To investigate whether the recorded Brillouin maps are susceptible to regional fluctuations in the refractive index (RI), RI tomographs were acquired for CM, DCM, and CU, (*N* = *2)*. The average RI for the CM was 1.438 ± 0.038 (ranging from 1.374 to 1.629), for the DCM it was 1.447 ± 0.070 (ranging from 1.356 to 1.663), and for the CU it was 1.426 ± 0.025 (ranging from 1.370 to 1.501). The observed reduction of RI was not statistically significant under Mann-Whitney-U test for CM-DCM and DCM-CU, but was found to be significant for CM-CU (*p* < *0.001*). The surface refractive index showed no structured variation when comparing periclinal and anticlinal regions (see Supplementary Fig. 8).

## Discussion

In BLS spectroscopy, the incident photon undergoes inelastic scattering with thermally induced density waves. The Doppler shift (BFS) induced by this process is proportional to the acoustic wave speed of the sample within the focal volume. The BFS (${\omega }_{B}$) for biological samples is in the order of 5-20 GHz and proportional to the acoustic speed (${v}_{a}$) within the focal volume^40^:

1 $${\omega }_{B}=\frac{2n{v}_{a}}{{\lambda }_{0}}\sin \left(\frac{\theta }{2}\right),$$

where $n$ corresponds to the refractive index (RI), ${\lambda }_{0}$ to the incident laser wavelength, and $\theta$ to the scattering angle. In backscattering geometry ($\theta$ = 180°), the triangular function is omitted. If the RI and the density of the sample ($\rho$) are known, the longitudinal modulus (storage modulus $M^{\prime}$) as a measure of compressibility of the sample can be calculated^38,40^:

2 $${M^{\prime}} =\rho \left(\frac{{\lambda \omega }_{B}}{{2n}}\right)^{2}.$$

In a similar manner, the imaginary part of the longitudinal modulus (loss modulus ${M}^{{{{\prime} }}{{{\prime} }}}$) can be calculated via the linewidth (Brillouin linewidth BLW, ${{{\Delta }}}_{B}$)^38,40,41^:

3 $${M}^{{{{\prime} }}{{{\prime} }}}={\left(\frac{{\lambda }}{2n}\right)}^{2}\rho {{\omega }_{B}}{{{{\Delta }}}_{B}}.$$

These equations show, that the longitudinal modulus is correlating with the BLS parameters by the factor $\rho /{n}^{2}$. This ratio is predicted to be approximately constant by the Lorentz-Lorenz equation, which was shown to hold true for hydrogels^41^ and some biological samples^42^, but only when no changes in polarizability occurs. However, the loss tangent $\tan \delta = {M^{\prime}} /{M^{\prime}}=4\pi {{{{\Delta }}}_{B}}/{{\omega }_{B}}$ relates the storage and loss module and is independent on RI and density. Thus, it provides a simple description for changes in viscoelastic properties independent of these quantities^38^. It is a measure of mechanical energy dissipation in the sample and thus a measure of relaxation/attenuation of mechanical waves in the time scale of picoseconds^35^.

Despite its significance in clinical translational research, the use of BLS in plant sciences has been very limited to date, primarily because expensive, optical equipment and well-trained operators are needed^40,43^. The BLS signal is weak compared to the commonly used Raman spectroscopy (Raman intensity approximately 10^5^ higher) and the BFS is small (approximately 5–20 GHz commonly observed in biological samples results in wavelength shifts below 1 cm^-1^). Consequently, high-contrast, low-loss, and highly dispersive spectrometers are necessary, i.e. for optical turbid samples a contrast of minimum 80 dB is required^44,45^.

In this study, BLS imaging was applied to hydrated, enzymatically isolated apple fruit CM as a model due to their biological stability. The surface scans allow a large field of view compared to cross-sectional images, which enables a statistical comparison of AR and PR, and can provide comparability to future in *vivo studies*. Further, the subsequent extraction of waxes and carbohydrates from the CM allowed to investigate their spatial mechanical impact on it.

Despite the chemical differences between CM, DCM, and CU, only a single Anti-Stokes/Stokes peak could be assigned to the cuticle. Consequently, the waxes and carbohydrates are smaller than the acoustic wavelength ${{\Lambda }}$ and therefore do not contribute to the spectrum directly^38^. It follows that the acoustic field experiences an effectively homogeneous medium. The BLS parameters, therefore, reflect the averaged mechanical properties of the chemical constituents.

Cuticular wax acts as a filler in the cutin matrix^21^ and fixes the growth-induced elastic strain of the CM^17,18^. Wax extraction results in a significant release of this fixed elastic strain^20^. Additionally, wax extraction significantly decreases the stiffness and fracture force of the CM^18^. This behavior was also observed in our study, represented by the BLS parameters (Fig. 1, Table 1). The BFS revealed a significant decrease from CM to DCM (Fig. 1b). This, in combination with non-changing RI (Supplementary Fig. 8) implies a decrease in acoustic wave speed.

As the extraction may change the polarizability, we cannot assume the Lorentz-Lorenz equation and therefore will refer to the loss tangent, which is independent of RI and density (Table 2). The averaged loss tangent was significantly reduced after dewaxing and indicates a decrease in attenuation of pressure waves and a decrease in strain in DCM compared to CM^23^. This observation demonstrates the change in mechanical behavior and is in line with previous macro-mechanical studies^18,26,46,47^.

The extraction of CHO resulted in a further significant reduction in stiffness, fracture force, and also a significant reduction in BFS compared to DCM (Fig. 1, Table 1). The loss tangent, however, increased for CU and was significantly higher compared to CM and DCM indicating a stronger attenuation of pressure waves. With the extraction of carbohydrates, the water uptake of the sample changes significantly^2,19,48^. Subsequently, the change in hydration of the CU impacts the measurement as well, as demonstrated in previous macro-mechanical experiments^17,27,49^. However, the finding underlines the critical role of polysaccharides in the mechanical properties of apple fruit CM.

Comparable results were reported for tomato fruit CM following CHO extraction^23,25^. It also aligns with the findings of the macro-mechanical uniaxial tensile test, where the removal of load-bearing components typically results in decreased stiffness. For instance, in cellulose composites, cellulose fibers serve as the primary load-bearing component^50^.

It is anticipated that trends in stiffness (as determined by tensile testing) and BLS parameters will be correlated. However, it is crucial to recognize that these mechanical properties cannot be directly compared quantitatively. This discrepancy arises from the fact that Brillouin spectroscopy probes the ratio of uniaxial stress to strain in a confined region and thus, allowing for a density and/or volume change^38^. In contrast, the uniaxial tensile test requires the volume to be kept constant. However, the observed reduction in BFS is noteworthy, particularly considering its magnitude (BFS = 0.9 GHz) compared to similar studies involving other plant samples. Previous research has demonstrated that even subtle BFS changes in plant samples can have pronounced effects on macro-mechanical behavior^30,32,35^.

In the heating and subsequently cooling experiments, the CM were subjected to temperature increases above the melting temperatures of the embedded wax (Fig. 2a, b), which were determined with DSC and cooled back to room temperature. This experimental approach induced a phase transition, reflected in the BLS signal and the DSC thermographs. Importantly, as waxes are hydrophobic and will not solve in the surrounding water, both the density and the RI did not change over the course of the experiment. Thus, all changes in BFS can be directly linked to the change of the longitudinal modulus (Eqs. 1 and 2). The observed hysteresis in the BFS (and thus *M*’) in the first heating and cooling cycle (Fig. 2a) can be attributed to the melting of the wax crystals and resulting relaxation of the cutin network during heating and the resolidification/vitrification of wax in its amorphous form (without additional dilation of the cutin network) during cooling^18^. The two-phase behavior observed in the heating period was similar to BLS studies involving pure paraffin (Supplementary Fig. 2), as reported in the literature^51–53^. However, the observed hysteresis and change in slope could suggest a significantly more complex phase transition, which requires further investigation.

The second temperature cycle showed significantly less hysteresis in the BLS signal and reduced heat flow, indicating that the wax remains amorphous in this short time frame. This finding is consistent with previous observations made using Fourier-domain infrared spectroscopy^18^. This underlines the importance of wax crystallinity for the strain fixation functions, likely via withstanding the lateral forces exposed to the CM. Heating the CM results in a loss of the crystal structure of the embedded wax and a partial release of elastic strain^18^. Consequently, the effect may be considered analogous to wax removal, albeit to a lesser extent due to the residual filling effect visible in the BLW (Fig. 2b and c).

Combining the findings prompts the hypothesis that the measured BLS signal predominantly captures the strain within the cutin-carbohydrate matrix, where the molecular chains of the cutin network exhibit a strain-stiffening effect. A similar strain-stiffening effect has been demonstrated using BLS spectroscopy for spider silk under external tensile load^54,55^.

In both isolated CM and DCM, the lateral BLS scans revealed a consistent micro-mechanical pattern mirroring the imprints of previously (before isolation) underlying epidermal cells (Fig. 3a)^56^. In CM, a significantly reduced BFS was observed in anticlinal regions (AR) in comparison to periclinal regions (PR) (Fig. 3b, d). However, the loss tangent was found to be lower in PR compared to AR (Table 2). As heating of the CM did not affect the BFS and loss tangent ratio (Supplementary Fig. 7), it is evident that, while the wax undergoes a change in state from crystalline to amorphous (Fig. 2), there is no redistribution of the waxes (Fig. 3b).

This implies (1) different acoustical wave speeds between AR and PR in the CM, indicating acoustical impedance mismatches between the regions, and (2) that waxes either contribute more to the mechanical properties of the cutin framework in the PR than in the AR and/or are not evenly distributed along the CM.

Upon wax removal, the AR exhibited a significantly higher BFS and loss tangent compared to the PR (Table 2). This reverses the AR/PR relationship observed in the BFS of CM (Fig. 3d) while the loss tangent ratio was not affected. The load-bearing structures in the DCM are mainly cellulose, hemicellulose, and pectin^2,22,57^, emerging from the epidermal cell walls and dominate the mechanical properties of the DCM^25,49,50,57^. Therefore, the observed spatial differences in BFS and BLW in the DCM suggests heterogeneity in the distribution of the crystallized carbohydrates as they are not directly contributing to the BLS signal. Supporting this hypothesis, the mechanical spatial heterogeneity disappeared in CU (Fig. 3).

The system used in this study was not sensitive to the anisotropy of biological samples. Thus, no evaluation of the anisotropy of strain was possible. However, the development of such a system was reported recently^37^.

By verifying our results with tensile testing and evaluating the possible influence of the RI, this study established Brillouin spectroscopy as a novel, noninvasive, and potentially in-vivo capable methodology enabling the measurement of mechanical properties, marking an important step regarding a deeper investigation of cuticle micro-mechanics. The results show that the cuticle cannot be regarded as a mechanically homogeneous construct, but has a pronounced micro-mechanical structure, which potentially has implications for the overall stability of the CM and the occurrence of microscopic defects.

## Materials and Methods

### Plant Material

Fruit of apple (*Malus × domestica* Borkh. cv Idared) were obtained from the Horticultural Research Station of the Leibniz University Hannover at Ruthe, Germany (lat. 52° 14’ N, long. 9° 49 ’E). Fruit trees were grown according to current EU regulations for integrated fruit production. Fruits free from visible surface blemishes were harvested at commercial maturity.

### Cuticle isolation, wax extraction, and carbohydrate extraction

CM samples were isolated enzymatically following the protocol described earlier^58,59^. For this, epidermal segments (ES) of 24 mm diameter were excised from the equatorial region of each fruit using a cork borer. Then, ES were incubated in 50 mM citric acid buffer solution (pH 4.0), containing pectinase (90 mL L^-1^; Panzym Super E liquid; Novozymes A/S, Krogshoejvej, Bagsvaerd, Denmark) and cellulase (5 mL L^-1^; Cellubrix L.; Novozymes A/S). To prevent microbial growth in the isolation medium, sodium azide (NaN_3_; 30 mM) was added. Isolation medium was refreshed 2 to 3 times until CM separated from the adhering tissue. Then, CM discs were cleaned using a soft brush, rinsed with deionized water, and dried. Wax was extracted in a Soxhlet apparatus using chloroform: methanol (1:1 v/v, 50 °C) for 2.5 h. Dried CM and dewaxed CM (DCM) were stored under ambient laboratory conditions until further use.

To extract the carbohydrates and remaining peptides, DCM samples were treated with 6 N HCl (Carl Roth, Karlsruhe, Germany) at 110 °C for 24 h^60,61^. Per batch, 15 - 25 DCMs were treated with a volume of 200 ml 6 N HCl in a 250 ml round neck flask with an attached reflux condenser. The extraction medium was gently stirred at 150 rpm to avoid mechanical disintegration of the DCM. The HCl-treated DCM were then taken out of the HCl, thoroughly washed with deionized water, and dried. The acid-resistant membranes obtained in this way consist mainly of cutin^46,47,60^ and are here after referred as CU.

### Brillouin light scattering micro-spectroscopy

**Fig. 4:**
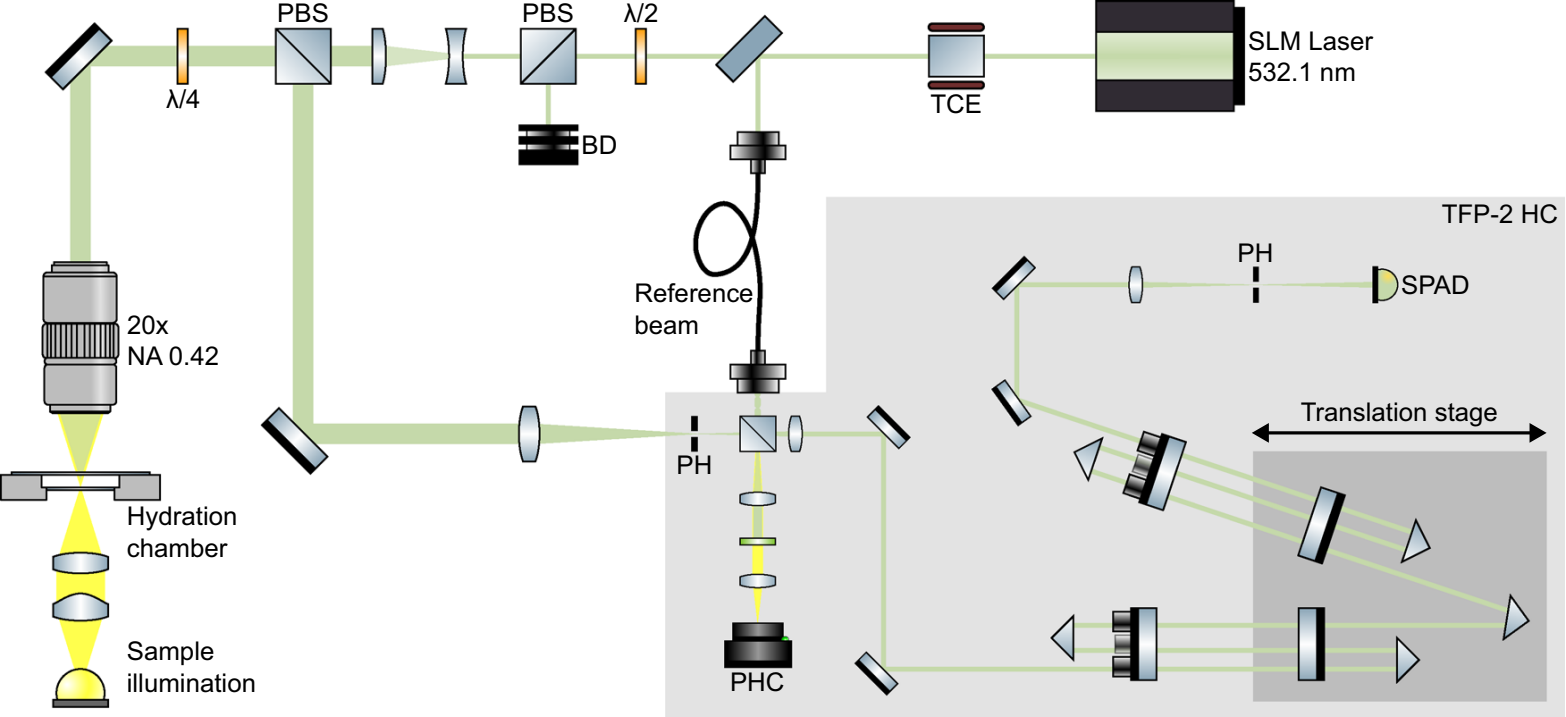
Schematic overview of the Brillouin light scattering micro-spectroscopy setup. The light from the single longitudinal mode (SLM) laser was further filtered by a temperature-controlled etalon (TCE) before passing power regulation optics (λ/2-plate and polarized beamsplitter; PBS) and entering the microscope. The sample is mounted in a hydration chamber and gets analyzed with circular polarized light (λ/4-plate). The Brillouin-scattered light gets collected in back-scattering geometry and analyzed with the commercially available 3 + 3-pass Tandem-Fabry-Pérot interferometer TFP-2 HC featuring two pinholes (PH), an entry-pinhole camera (PHC) and a single photon avalanche diode (SPAD).

A 1 MHz linewidth single longitudinal mode laser with a wavelength of 532.1 nm (Samba; Cobolt AB, Solna, Sweden) was coupled into a custom build automated microscope^36^ (Fig. 4). The laser spot was focused using a long working distance 20x objective (M Plan Apo; Mitutoyo, Takatsu-ku, Japan) with a numerical aperture (NA) of 0.42, resulting in a theoretical diffraction-limited $1/{e}^{2}$ spot diameter of approximately 770 nm laterally. The same objective was used to collect the back-scattered light. Prior to reaching the objective, circularly polarized light was achieved by introducing a λ/4 plate upstream in the optical path, effectively illuminating the sample with circular polarized light. The laser power on the sample was 0.76 mW. The scattered light was then directed back through a polarized beamsplitter to separate it for analysis with the Brillouin-spectrometer. The Brillouin-scattered light was analyzed using the 3 + 3-pass Tandem Fabry-Pérot interferometer (TFP-2 HC; The Table Stable Ltd., Mettmenstetten, Switzerland). The spectrometer was actively stabilized during each scan to the laser wavelength, enabling prolonged acquisition without any loss of frequency lock. The TFP features two selectable pinholes. The first limits the entering light, allowing a semi-confocal configuration, while the exit pinhole sets the finesse of the Fabry-Pérot interferometer for a given mirror spacing. The entry pinhole was 600 µm. The exit pinhole was set to 450 µm resulting in a finesse of approx. 80 for the set mirror spacing of 3 mm.

Prior to BLS scanning, the CM, DCM, and CU discs were rehydrated in deionized water for at least 16 h and visually inspected for defects. Samples were sandwiched between two coverslips and mounted in a hydration chamber with a see-through top window. This was crucial to keep the sample fully hydrated for prolonged acquisition times. Additionally, the water served as heat conducting medium for the generated heat of the laser. Therefore, combined with the low laser power, the heat contribution under these conditions was estimated to be negligible.

The effect of exposing CM to various temperature on micro-mechanical properties was studied by heating the hydration chamber from the bottom, using a custom developed heat plate, which was controlled via a micro-controller and allowed for continuous heating of the sample up to 100 °C by Peltier elements.

For lateral scanning purposes, the hydration chamber and heating plate were mounted onto a motorized lateral scanning stage (MLS203-1; Thorlabs Inc., Newton, USA), enabling precise and controlled movement during the acquisition. The scanned region was 120 x 120 µm^2^ with a 4 µm step width, respectively. Focusing was accomplished by moving the objective using a vertical scanning stage (PLSZ; Thorlabs Inc., Newton, USA).

Synchronization of the stage movements and the start of each acquisition was handled by a custom python script. The detector integration time per pixel was set to 25.6 s.

#### Data Processing

The acquired Brillouin data was analyzed using a custom-written python script. First, the acquired deconvolved using the automatically detected instrumental response function. The partly overlapping spectral contributions of the sample and deionized water were spectrally unmixed using two instances of power spectral density functions of the damped harmonic oscillator (DHO) as in Eq. 4^62^.

4 $${I}_{{\mbox{DHO}}}\left(\omega ,\,{I}_{0},{\omega }_{B}{,{{\Delta }}}_{B},{B}\right)=\frac{1}{\pi }\frac{{I}_{0}4{\omega }_{B}^{2}{{{\Delta }}}_{B}}{{\left({\omega }^{2}-{\omega }_{B}^{2}\right)}^{2}+4{\omega }_{B}^{2}{{{\Delta }}}_{B}^{2}}+B$$

where$\quad {I}_{0}$ denotes the Brillouin scattering intensity, ${\omega }_{B}$ the Brillouin frequency shift (BFS), ${{{\Delta }}}_{B}$ the corresponding peak half width (Brillouin line width; BLW), and $B$ a common baseline mainly representing the detector dark count rate. Commonly, the signal showed the centered elastically scattered Rayleigh peak and two pairs of symmetric peaks corresponding to water and the cuticle.

#### Separation of anticlinal and periclinal signals

To distinguish the signals of anticlinal regions (AR) and periclinal regions (PR) of the acquired lateral cuticle maps, an adaptive threshold was utilized. The cuticle Brillouin signal was fitted as described in the section above and further normalized using the water Brillouin signal. As the AR tend to be thicker^3,63^, they cover a larger proportion of the focal volume. This generates a stronger Brillouin signal. By calculating the ratio, we can obtain information about the peak prominence, from which a relative thickness map was derived for the scanned area and verified using brightfield microcopy^56^. The adaptive threshold was determined using the weighted sum of the neighborhood pixel values, separating the anticlinal and periclinal regions of the cuticle map by the relative thickness.

### Uniaxial tensile test

Macro-mechanical properties of the CM, DCM and CU were quantified using a standard uniaxial tensile test following the procedure described in earlier work^18,26^. In brief, 5 mm wide strips of CM, DCM or CU were excised using parallel-mounted razor blades. Subsequently, these strips were mounted in frames made of masking tape (Tesa Krepp; tesa Werk Hamburg GmbH, Hamburg, Germany) and paper. The strips, including the paper frame were then hydrated in deionized water for 16 hours and subsequently, the frames were secured between the clamps of a universal material testing machine (Z 0.5; Zwick/Roell, Ulm, Germany) equipped with a 10 N standard load cell (KAP-Z; Zwick/Roell). The clamping distance (${L}_{0}$) was set to 10 mm.

Thereafter, the paper frames were cut open and uniaxial tensile force was applied (crosshead speed 1 mm min^-1^) continuously until specimen failure occurred. Throughout the test, both the applied force ($F$) and the specimen length ($L$) were continuously recorded. Data for specimens that failed within or adjacent to the clamps and those exhibiting irregular stress-strain curves were excluded from the analysis, as these anomalies may have been caused by handling or mounting issues. The maximum force (${F}_{\max }$ in N) required to break the specimen closely resembles the force at failure. Strain (*ε* in %) was calculated using the following formula:

5 $$\varepsilon =\frac{L-{L}_{0}}{{L}_{0}}\times 100$$

The stiffness (*S* in ${\mbox{N}}$) of the specimen was calculated as the maximum slope of a linear regression fitted to the force-strain curves.

Due to the irregular nature of the cross-sectional surface of CM, the calculation of stress (force per unit cross-sectional surface area) is not realistic. Thus, determination of the more commonly used Young’s modulus was not possible.

### Refractive index tomography

Refractive index tomographs of cross-sections of CM, DCM, and CU were acquired. For this, the samples were embedded in CryoGlue (SLEE medical GmbH, Nieder-Olm, Germany) and quickly frozen using liquid nitrogen. Subsequently, 10 µm thick cross-sectional slices were prepared using a cryotome (MEV; SLEE medical GmbH, Nieder-Olm, Germany). After cutting, the slices were rinsed in deionized water and kept hydrated until measurement. The acquisition was done using a holographic phase microscope with a rotating scanner (3D Cell Explorer; Nanolive SA, Lausanne, Switzerland). The microscope was equipped with a 60x objective (BE Plan 60x, Nikon, Tokyo, Japan) with a NA of 0.8. Tomographs were evaluated using the maximum intensity projection perpendicular to the surface.

### Differential scanning calorimetry

The CM and DCM samples were additionally analyzed using differential scanning calorimetry (DSC) to monitor phase transitions of the wax as affected by heating. The samples were punched out with a biopsy punch (SMI AG, St.Vith, Belgium) with a diameter of 4 mm and weighted (mean weight 375 µg) into standard 40 µl aluminum sample pans and the pans were crimped. Samples were loaded in the DSC (DSC1 StarE; Mettler Toledo, Columbus, Ohio, USA) and subsequently heated/cooled from 25 °C to 125 °C with a set heating/cooling rate of 10 °C/min. Samples were held at maximum temperature for 10 min.

### Statistics and reproducibility

18 Isolated CM were grouped into CM, DCM, and CU. DCM and CU were treated accordingly. Each group contained 6 technical replicates originating from different apples and trees.

The statistical analysis of repeated measurements taken at different temperatures and during distinct temperature phases was conducted using the R software environment. The measurements in both phases were characterized by non-equidistant temperature intervals. To facilitate phase comparison, the data were interpolated accordingly: linear interpolation was applied to the BFS data, while quadratic interpolation was utilized for the BLW data.

For the modeling process, numerous linear mixed-effect models were constructed using the *nlme* package^64^. These models incorporated various covariance structures, including scaled identity, first-order autoregression, first-order heterogeneous autoregressive, compound symmetry, Toeplitz, and heterogeneous Toeplitz. The best-performing model (heterogeneous Toeplitz with additional random scanning position) was chosen according to the Akaike Information Criterion^65^, Bayesian information criterion^66^ and its maximum logarithmic likelihood. Post-hoc analysis was performed using Tukey’s HSD test at *p* < 0.05 to visualize significances between heating and cooling phases.

### Reporting summary

Further information on research design is available in the Nature Portfolio Reporting Summary linked to this article.

## Supplementary information

Supplementary information

Reporting Summary

## Data Availability

The underlying data for all the primary and Supplementary Figs. has been deposited in a publicly accessible repository [10.25835/xvsi5g6m]^67^. Raw data may be obtained from the authors upon reasonable request.

## Code availability

The python script for Brillouin data analysis may be obtained from the authors upon reasonable request.
