# Supplementary material for: Micromechanical behavior of the apple fruit cuticle investigated by Brillouin light scattering microscopy
Source: Commun Biol. 2025 Feb 4;8:174. doi: 10.1038/s42003-025-07555-5 (PMC11794438; doi:10.1038/s42003-025-07555-5)
Supplement: Supplementary file 1 — Supplementary information [file 42003_2025_7555_MOESM1_ESM.pdf]

Supplementary Information:

Micromechanical behavior of the apple fruit cuticle investigated by Brillouin light scattering microscopy

Timm Landes[1,2,3], Bishnu Prasad Khanal[1,3], Hans Bethge[1,3], Tina Lehrich[4], Maximilian Seydi Kilic[5], Franz Renz[5], Miroslav Zabic[1,3], Moritz Knoche[3], Dag Heinemann[1,2,3,*]

1. Hannover Centre for Optical Technologies, Leibniz University Hannover, Nienburger Straße 17, 30167 Hannover, Germany
2. Cluster of Excellence PhoenixD, Leibniz University Hannover, Welfengarten 1a, 30167 Hannover, Germany
3. Institute of Horticultural Production Systems, Leibniz University Hannover, Herrenhäuser Straße 2, 30419 Hannover, Germany
4. Institute of Cell Biology and Biophysics, Leibniz University Hannover, Herrenhäuser Straße 2, 30419 Hannover, Germany
5. Institute of Inorganic Chemistry, Leibniz University Hannover, Callinstraße 3-9, 30167 Hannover, Germany

*** Author for Correspondence:** Dag Heinemann: dag.heinemann@hot.uni-hannover.de

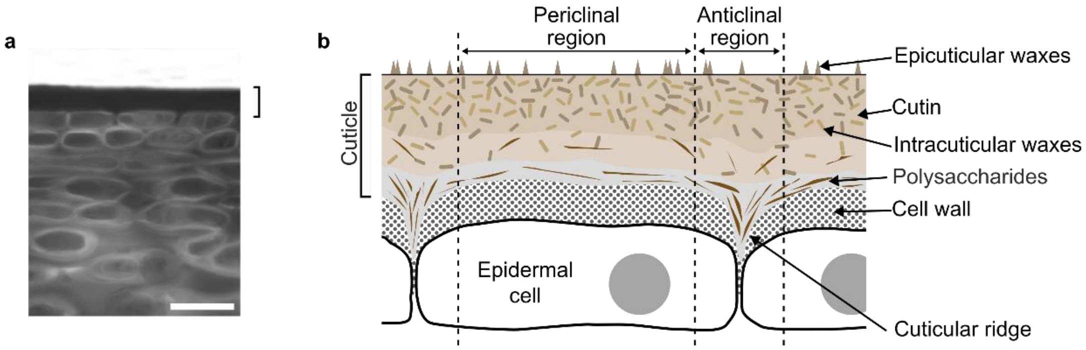

Supplementary Figure 1. Cross-sectional overview of the apple fruit cuticular membrane. (a) Microscopic image of apple (cv. Idared) fruit skin cross-section. (b) Schematic drawing of cuticle including outer epidermal cell wall, showing distribution of different components (cutin, wax, polysaccharides) and the two distinct regions (periclinal and anticlinal) of the cuticle. Scale bar in (a) equal 25 µm.

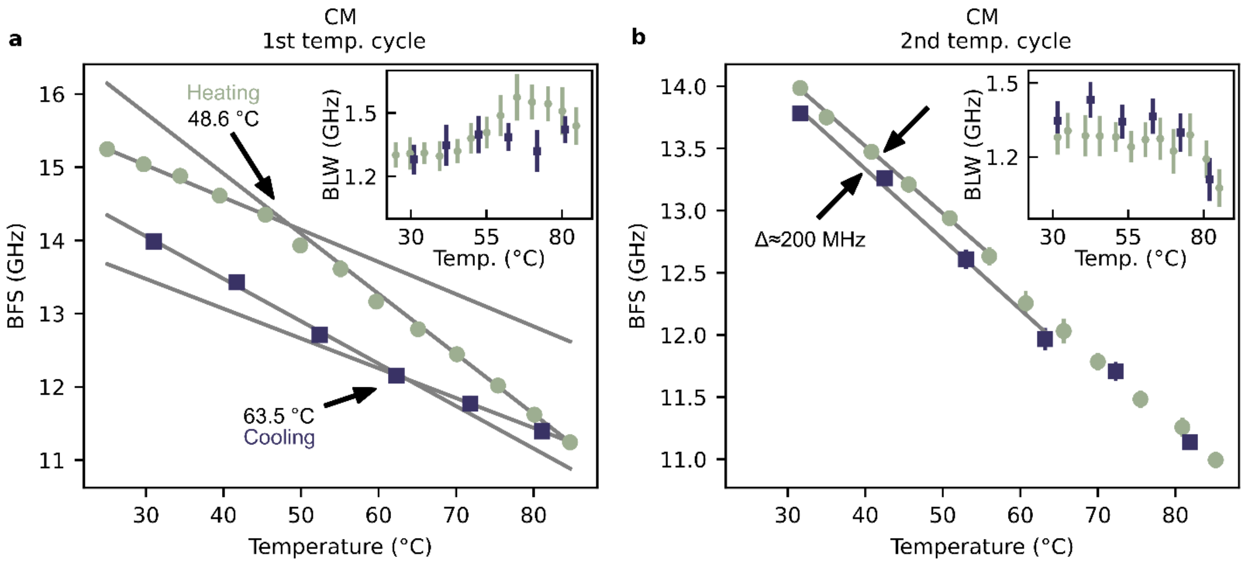

Supplementary Figure 2. Detailed analysis of the temperature hysteresis for untreated cuticular membrane (CM). (a) Brillouin frequency shift (BFS) for the first heating and cooling phase. The slope changed for heating at approximately 48.6 °C and for cooling at 63.5 °C. The temperature offset to the DSC data (51 °C and 67 °C) results from the heating of the laser. The inset shows the Brillouin line width (BLW) for reference. (b) BFS for the second temperature cycle of the same CM. The hysteresis occurred only at temperatures below 70 °C and was significantly reduced. The inset shows the BLW for reference.

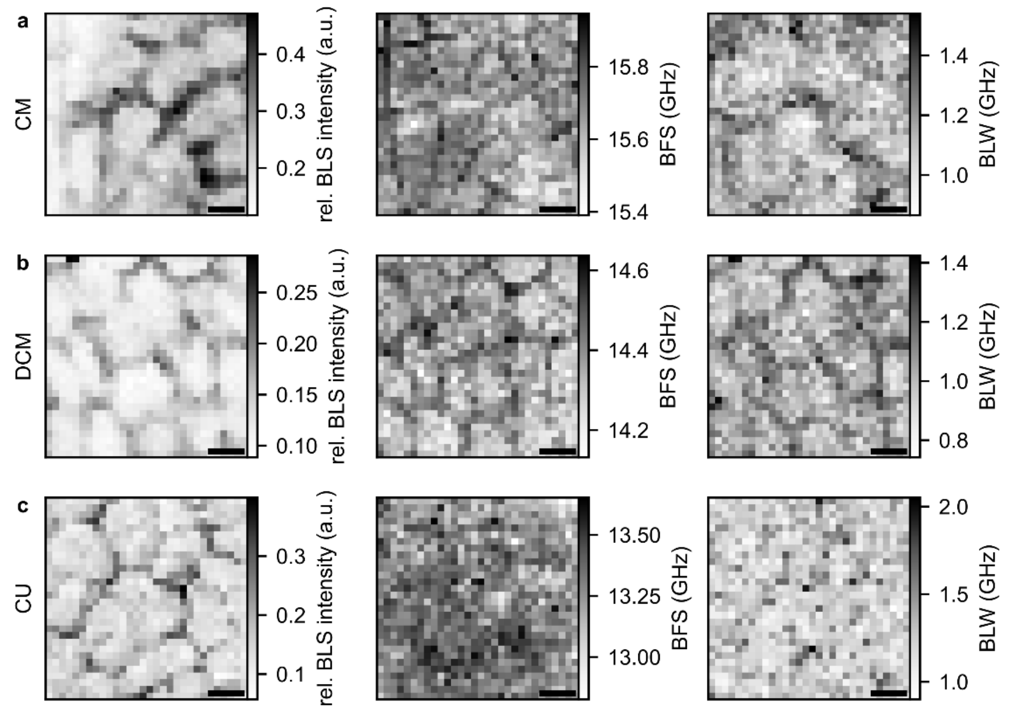

Supplementary Figure 3. Representative Brillouin spectroscopy maps for the samples. Relative Brillouin light scattering (BLS) intensity of sample peak normalized to the water peak, Brillouin frequency shift (BFS), and Brillouin linewidth (BLW) for (a) native cuticular membrane (CM), (b) dewaxed CM (DCM), and (c) hydrochloric acid treated DCM (CU). Scale bars denote 20 μm.

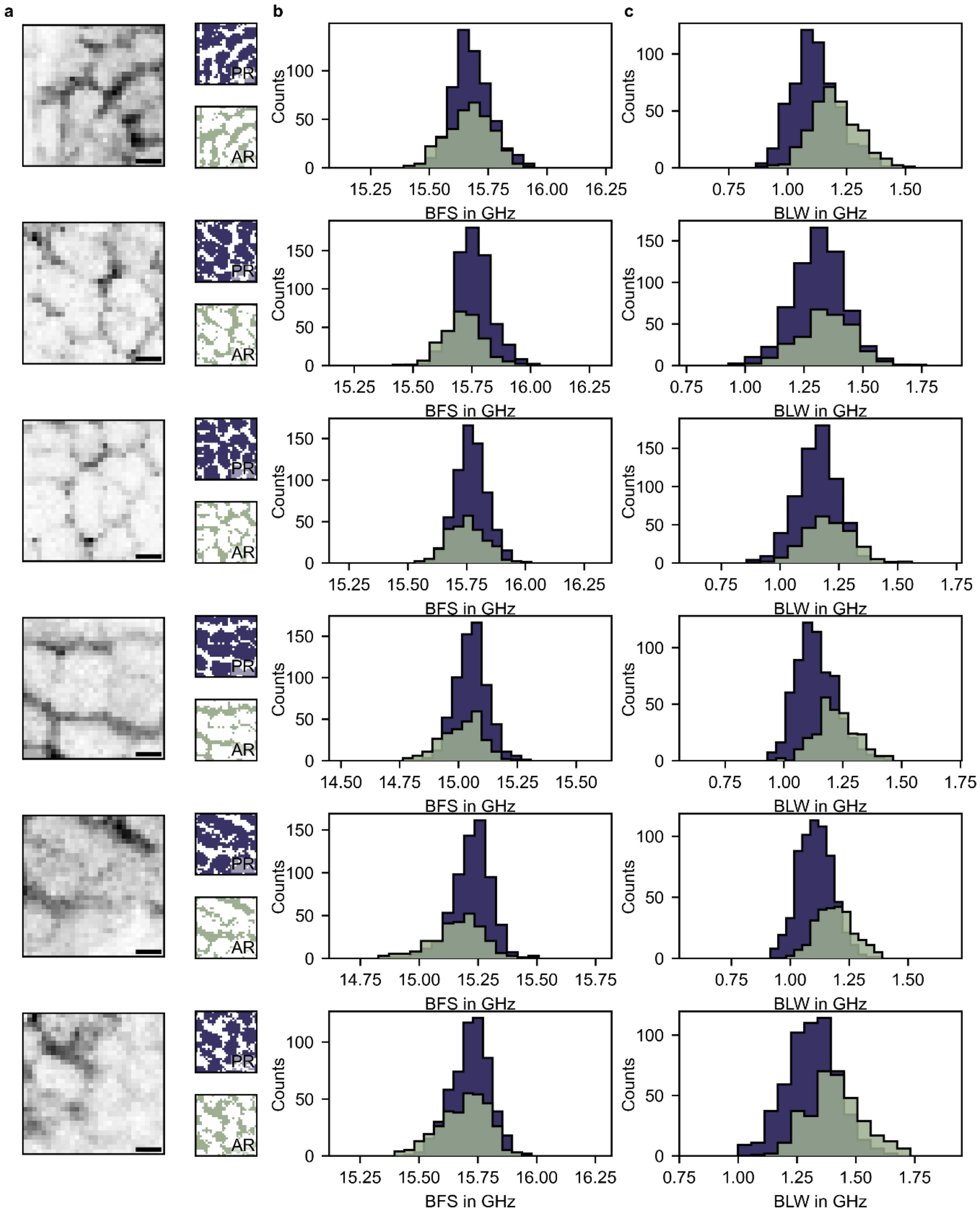

Supplementary Figure 4. Thresholded masks of isolated cuticular membranes (CM). (a) Brillouin intensity maps of cuticular membranes and calculated masks of the periclinal regions (PR) and anticlinal regions (AR). (b) Brillouin frequency shift (BFS) and (c) Brillouin line width (BLW) histograms for the PR and AR are evaluated using the respective mask. Scale bars denote 20 µm. Areas out of focus were excluded from further analysis.

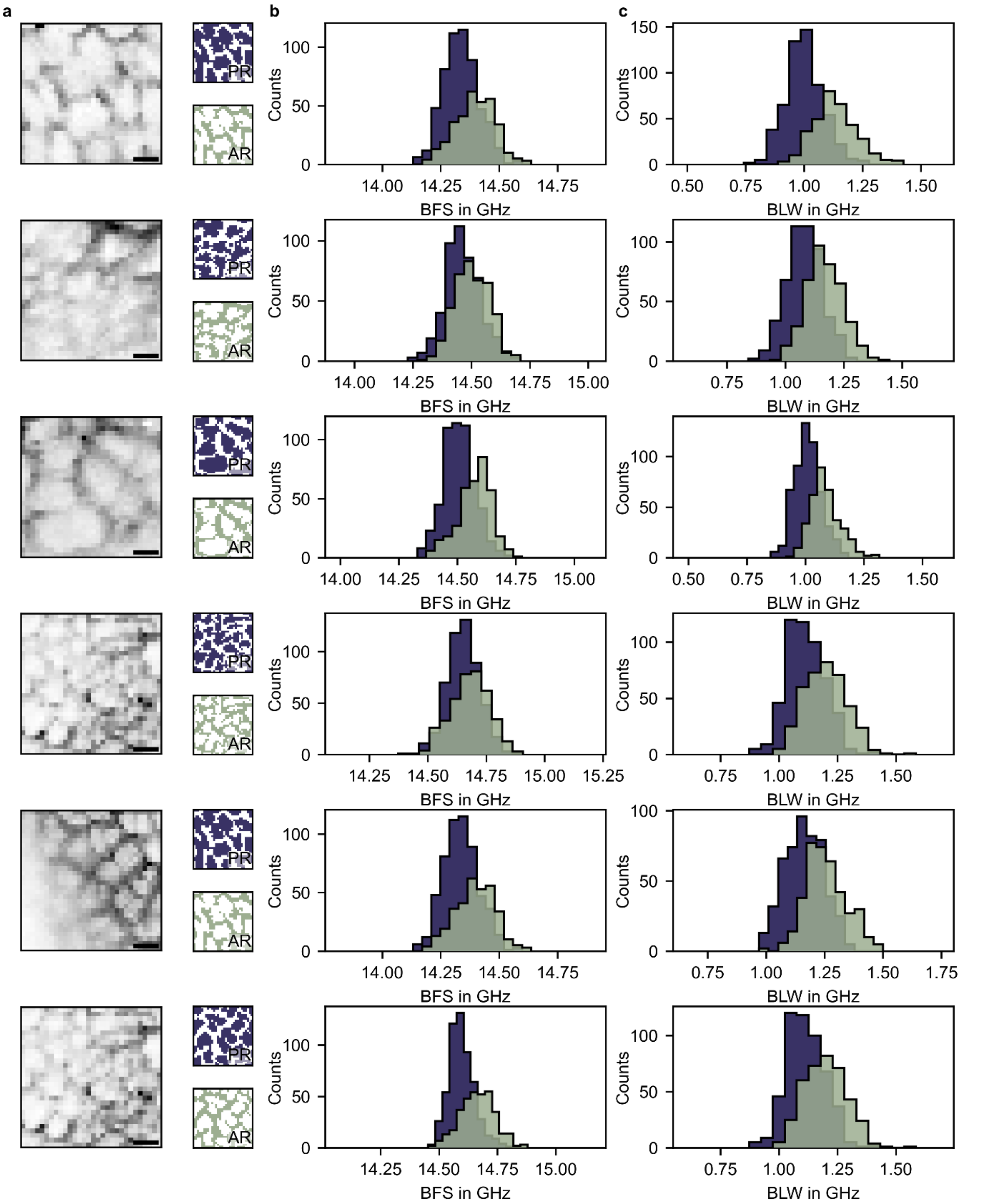

Supplementary Figure 5. Thresholded masks of isolated dewaxed cuticular membranes (DCM). (a) Brillouin intensity maps of dewaxed cuticular membranes and calculated masks of the periclinal regions (PR) and anticlinal regions (AR). (b) Brillouin frequency shift (BFS) and (c) Brillouin line width (BLW) histograms for the PR and AR are evaluated using the respective mask. Scale bars denote 20 µm. Areas out of focus were excluded from further analysis.

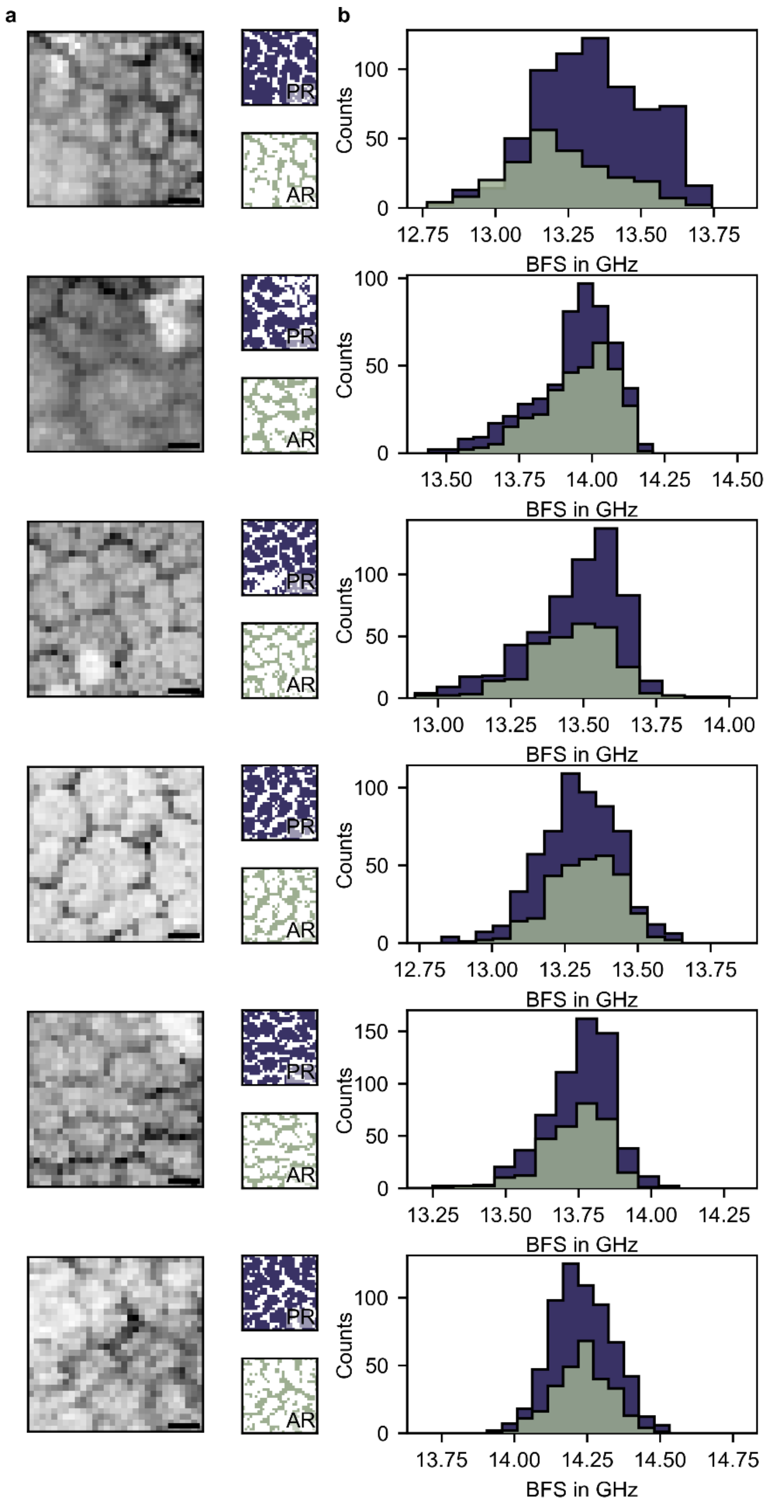

Supplementary Figure 6. Thresholded masks of HCl treated isolated dewaxed cuticular membranes (CU). (a) Brillouin intensity maps of dewaxed and HCl treated cuticular membranes and calculated masks of the periclinal regions (PR) and anticlinal regions (AR). (b) Brillouin frequency shift (BFS) and (c) Brillouin line width (BLW) histograms for the PR and AR are evaluated using the respective mask. Scale bars denote 20 µm. Areas out of focus were excluded from further analysis.

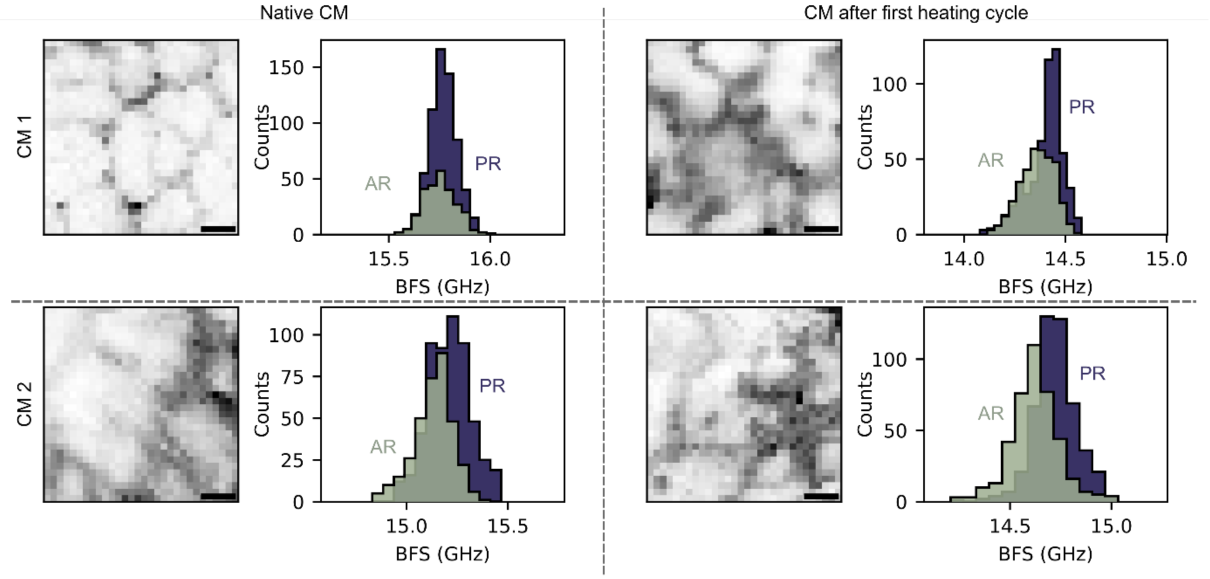

Supplementary Figure 7. Impact of heating on isolated apple cuticle and the mechanical heterogeneities. Intensity signals and Brillouin frequency shift (BFS) locally thresholded for anticlinal regions (AR) and periclinal regions (PR) of ‘Idared’ apple cuticular membrane (CM) before heating and after heating to 90 °C and subsequent cooling to room temperature. Scale bars denote 20 µm.

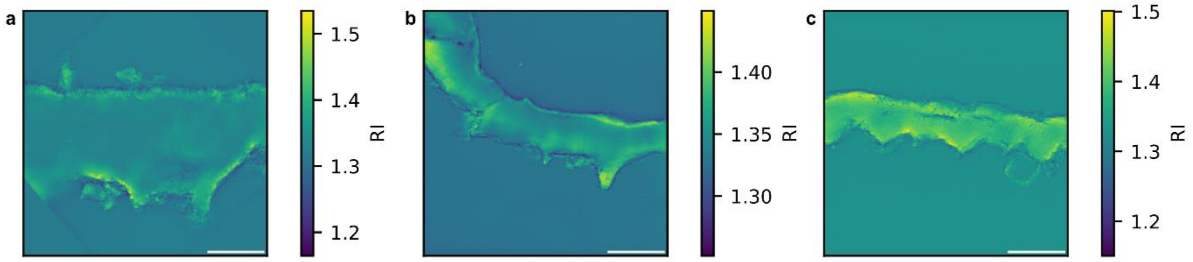

Supplementary Figure 8. Refractive index tomographs of isolated apple cuticle samples. Refractive Index (RI) tomographs for the isolated apple fruit cuticular membrane (a), the dewaxed cuticular membrane (b) and the HCl treated dewaxed cuticular membrane (c). Scale bars denote 20 µm.
